# Permanent neurological morbidity after microsurgical resection of hereditary hemorrhagic telangiectasia-associated brain arteriovenous malformations: a systematic review and illustrative case

**DOI:** 10.1016/j.bas.2026.107455

**Published:** 2026-09-22

**Authors:** Ján Buvala, Christopher S. Ogilvy, Juraj Šteňo, Juraj Klepoch, Vladimír Javorka, Michaela Jezberová, Peter Jankovič, Parvez M. Shaikh, Andrej Šteňo

**Affiliations:** a Department of Neurosurgery, Faculty of Medicine of Comenius University and University Hospital Bratislava, Limbová 5, Bratislava, 83305, Slovakia; b Neurosurgical Service, Beth Israel Deaconess Medical Center, Harvard Medical School, 110 Francis St., Boston, MA, 02215, United States; c Clinical Neuroscience Research Unit, Department of Neurosurgery, Faculty of Medicine of Comenius University and University Hospital Bratislava, Limbová 5, Bratislava, 83305, Slovakia; d Department of Radiology, Faculty of Medicine of Comenius University and University Hospital Bratislava, Limbová 5, Bratislava, 83305, Slovakia; e Dr. Magnet Kramáre, Dr. Magnet s.r.o., Limbová 5, Bratislava, 83305, Slovakia; f Department of Neurosurgery, F.D. Roosevelt University Hospital with Policlinic, Nám. L. Svobodu 1, Banská Bystrica, 97517, Slovakia; g Department of Radiation Oncology, West Virginia University, PO Box 9234, 44 Medical Center Drive, Morgantown, WV, 26506-9234, United States

**Keywords:** Brain arteriovenous malformation, Rendu-Osler-Weber disease, Hereditary hemorrhagic telangiectasia, Surgical outcome, Neurological morbidity, Systematic review

## Abstract

**Introduction:**

The optimal management of brain arteriovenous malformations (AVMs) in patients with hereditary hemorrhagic telangiectasia (HHT) remains controversial. Some authors recommend conservative management based on the presumed lower rupture risk compared to sporadic AVMs, others advocate surgical treatment. No systematic review has focused on permanent neurological morbidity following resection of HHT-associated AVMs, leaving a significant gap in the literature.

**Research question:**

Do the unique genetic features and angio-architecture of HHT-associated AVMs result in a different profile of postoperative neurological morbidity compared to sporadic AVMs?

**Materials and methods:**

A systematic search of PubMed, Embase, and Cochrane databases (1947–April 2026) was conducted per PRISMA 2020 guidelines; 18 publications reporting surgically treated HHT-associated brain AVMs were identified, 12 were included in the comparative analysis. Study quality was assessed using the Joanna Briggs Institute Critical Appraisal Checklist. Permanent morbidity rates were compared with unselected sporadic AVM series using Fisher's exact test with sensitivity analyses.

**Results:**

Among 91 patients, pooled incidence of new permanent neurological deficits was 9.9% (95% CI: 4.6–18.0%). No significant difference was found compared to unselected sporadic AVM cohorts. One study contributed 78% of permanent deficit events; its exclusion reduced the rate to 2.9%. No postoperative mortality was reported.

**Discussion and conclusion:**

No significant difference in permanent neurological morbidity was demonstrated between HHT-associated and sporadic AVM resections. However, the small sample size precludes definitive conclusions. These hypothesis-generating findings may aid in individualized risk assessment and shared decision-making. An illustrative case is presented to demonstrate the clinical context motivating this review.

## Highlights

• First systematic review of surgical outcomes in HHT-associated brain AVMs.
• Pooled new permanent neurological morbidity after resection was 9.9%.
• No statistical difference vs. sporadic unselected brain AVM surgery outcomes.
• Brain AVM resection may be a reasonable option in carefully selected HHT patients.
• Small sample size precludes firm conclusions, findings are hypothesis-generating.

## 1 Introduction

The management of hereditary hemorrhagic telangiectasia (HHT; i.e., Rendu-Osler-Weber disease)-associated brain arteriovenous malformation (AVMs) remains controversial [Meybodi et al., 2018; Frantzias et al., 2024; Faughnan et al., 2020; Derdeyn et al., 2017]. The usual risk stratification for surgery of HHT-associated AVMs - age, location of lesion, arterial and venous anatomy - does not appear to differ from sporadic AVMs. However, some authors recommend conservative management of HHT-associated AVMs based on the presumed lower rate of hemorrhages compared to sporadic brain AVMs [Yang et al., 2016; Willemse et al., 2000]. In contrast, other authors doubt their indolent nature and advocate an active treatment of HHT-associated AVMs [Gamboa et al., 2018; Woodall et al., 2019]. Interestingly, to the best of our knowledge no systematic review on permanent neurological deficits of resections of HHT-associated brain AVMs was published. A systematic review could clarify whether the unique genetic features and angio-architecture of HHT-associated AVMs result in different profiles of postoperative neurological morbidity compared to sporadic AVMs, which is currently unknown. The clinical urgency of this question is especially prominent in patients who decline stereotactic radiosurgery because of its latency period and request immediate definitive treatment; for such patients, reliable data on surgical morbidity are essential for informed shared decision-making. Considering the generally challenging management of HHT patients and the high level of their natural disease-related anxiety [Chaturvedi et al., 2017], detailed patient information regarding the benefits and risks of treatment of HHT-associated brain AVMs is of particular importance, and relevant data are therefore needed. An illustrative case demonstrating this clinical scenario is presented alongside the systematic review.

The aim of this paper is to provide up-to-date information on functional outcome of resections of HHT-associated brain AVMs based on a systematic literature review with a pooled data analysis.

## 2 Illustrative case description

A 49-year-old right-handed male smoker with arterial hypertension; history of intermittent headache, recurrent collapses, lassitude, recurrent melena and epistaxis. Admitted for collapsing due to severe anemia in July 2022. The patient was diagnosed with multiple telangiectasias on the face and multiple AVMs – in the lungs (bilaterally), nasal cavity and brain. Patient was diagnosed with hereditary hemorrhagic telangiectasia, Curacao criteria 4/4, neurological investigation was normal, speech and language investigation revealed no deficits. Digital subtraction angiography (DSA) (Fig. 1A and B) and 3 T Magnetic Resonance Angiography (3 T MRA) visualized an AVM in the dominant left frontal operculum, the AVM was scored as Spetzler-Martin grade II. Magnetic Resonance tractography showed close relationship of AVM to left arcuate fasciculus (Fig. 1C). After a detailed explanation of the treatment options, the patient declined stereotactic radiosurgery due to its delayed effect and personal experience with the death of his father, who also suffered from HHT and who died as a result of intracranial hemorrhage, and requested a faster treatment option. Both the neurosurgeon and the interventional radiologist preferred surgical AVM resection to endovascular treatment. A left frontotemporal craniotomy was performed and a standard microsurgical resection of AVM with indocyanine green (ICG) was performed. Navigated 3D-US power-Doppler was used in order to compensate for the brain shift [Mathiesen et al., 2007; Unsgård et al., 2016] (i.e. to refine navigation accuracy) and to verify the presence/absence of pathological vascular signal after AVM resection (Fig. 1.) as reported before by other groups [Carl et al., 2018; Walkden et al., 2015]. Postoperative computed tomography angiography (CTA) and MRA showed complete AVM resection. Mild transient postoperative aphasia fully recovered one month after the surgery as verified by detailed speech and language investigation.

**Fig. 1:**
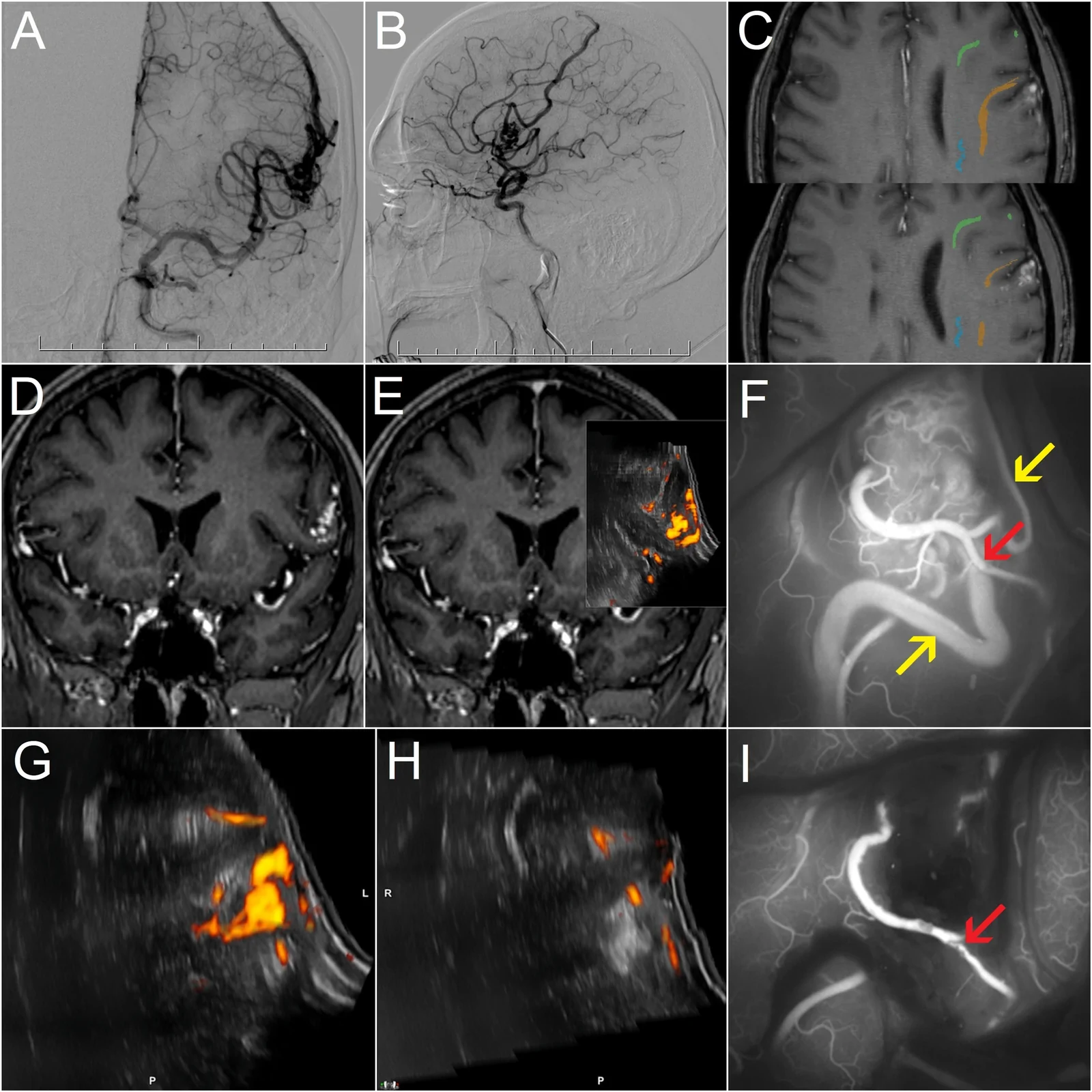
Images obtained during the therapeutic management of the illustrative patient. A and B: DSA showing small AVM in the left frontal operculum. The AVM nidus was in close proximity to the arcuate fasciculus (orange) as demonstrated by the preoperative MRI tractography (C); green = fasciculus fronto-occipitalis inferior, blue = pyramidal tract. D: preoperative contrast enhanced T1-weighted MRI navigation sequence (coronal scan) and the same MRI sequence fused with the intraoperative 3D-ultrasound power-Doppler before the AVM resection (E); note the good correspondence between MRI and 3D-ultrasound image. F: ICG video angiography before AVM resection; red arrow = “en passage” artery supplying eloquent brain; yellow arrows = AVM draining veins. G + H: preresectional and postresectional 3D-ultrasound power-Doppler image rendered in axial plane; no residual AVM vessels are visible after the resection. I: ICG video angiography after AVM resection; no residual AVM vessels were visible; red arrow = preserved “en passage” artery after disconnection of feeders.

## 3 Materials and methods

### 3.1 Study design and research question

This study was designed as a systematic review with pooled descriptive analysis, supplemented by an illustrative case demonstrating the specific clinical context. The primary research question was structured according to the PICO framework:

- **Population**: Patients with hereditary hemorrhagic telangiectasia (HHT) harboring brain arteriovenous malformations (AVMs)
- **Intervention**: Microsurgical resection of brain AVMs
- **Comparator**: Published benchmark rates of permanent neurological morbidity after microsurgical resection of sporadic (non-HHT) brain AVMs
- **Outcome**: New permanent postoperative neurological deficits. Permanent neurological morbidity was defined as any new neurological deficit or functional worsening persisting at last follow-up.

This framework guided the literature search strategy, inclusion criteria, data extraction, and statistical comparison. The review protocol was not prospectively registered in PROSPERO. The methods, including the search strategy, eligibility criteria, quality assessment, and planned analyses, are reported in full below in accordance with PRISMA 2020.

### 3.2 Literature review

We performed a literature search of published articles using PubMed, Embase and Cochrane database to identify articles published from 1947 to April 2026 with the key words “(Rendu OR hereditary hemorrhagic telangiectasia) AND (cerebral OR brain OR central nervous system) AND (arteriovenous OR AVM)” according to the PRISMA 2020 guidelines. After identifying and removing duplicate records, we identified 1761 publications, from which we selected 18 publications directly addressing surgical treatment of cerebral AVMs in HHT patients; only English-language articles were selected (Fig. 2).

**Fig. 2:**
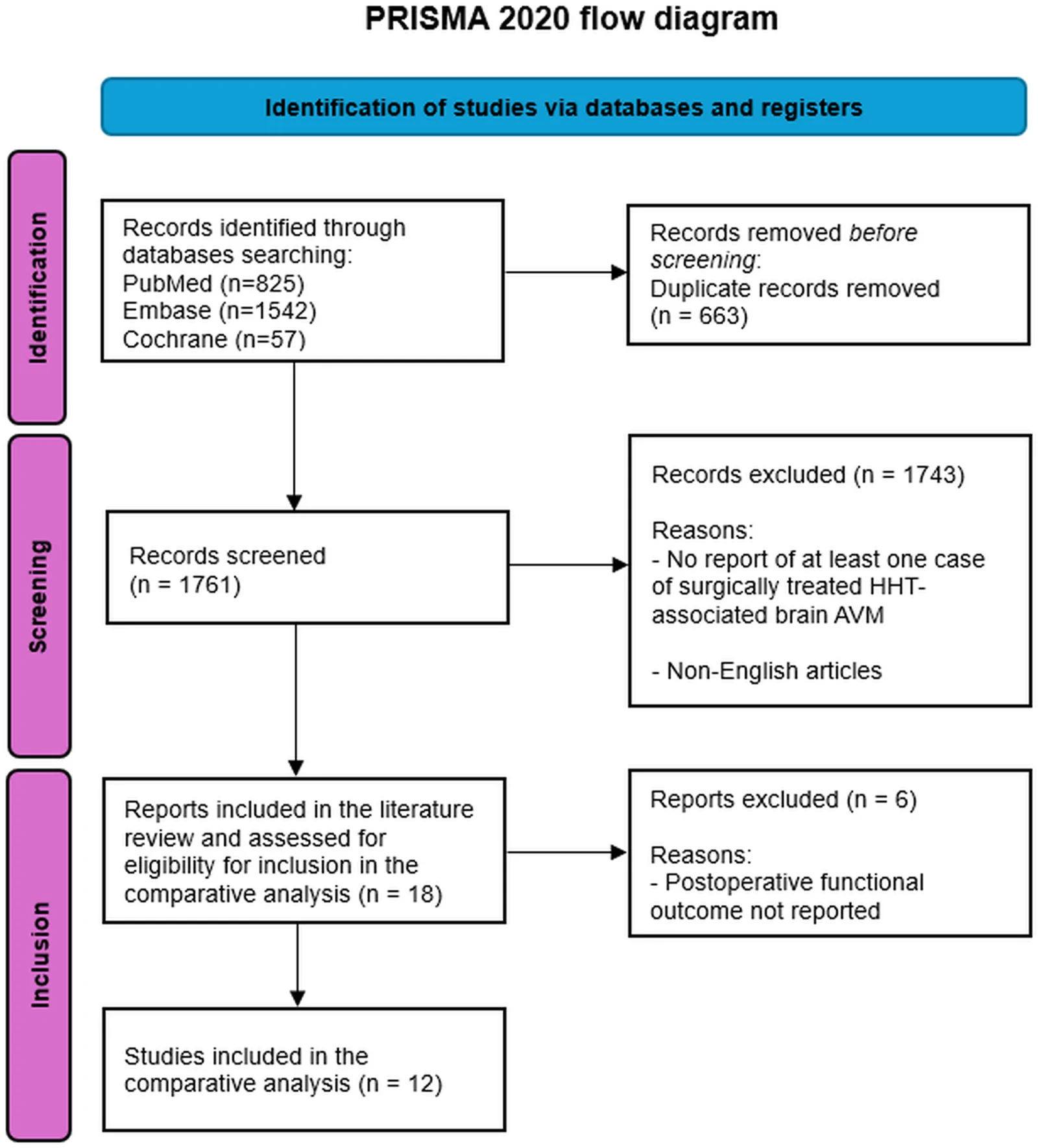
PRISMA diagram showing the methods for the selection of the studies included in the review and the analysis.

In order to compare the incidence of permanent neurological morbidity after resections of HHT-associated brain AVMs with the incidence of permanent neurological complications after resections of sporadic brain AVMs, we performed another literature research of published articles using PubMed with the key words “intracranial AND arteriovenous AND surgery AND deficit” to identify relevant literature on surgical treatment of sporadic brain AVMs and on its outcome.

The methodological quality of included studies was assessed using the Joanna Briggs Institute (JBI) Critical Appraisal Checklist for Case Series [Aromataris et al., 2024] a validated 10-item tool evaluating inclusion criteria, condition measurement, consecutive and complete inclusion of participants, demographic and clinical reporting, outcome reporting, site identification, and statistical appropriateness. Each item was rated as “Yes,” “No,” “Unclear,” or “Not applicable.” To quantify methodological quality, a modification of JBI score was used, in which the percentage represents the ratio of “Yes” responses to the total number of items on the relevant checklist (including “N/A″ responses). This approach was chosen to prevent artificially inflating scores in studies with a high number of irrelevant categories. Studies scoring ≥8/10 were classified as high quality, those scoring 7/10 as moderate quality, and those scoring ≤6/10 as low quality. The assessment was performed independently by two authors (J.B. and A.Š.), with disagreements resolved by consensus. Results are presented in Supplementary Table 1.

### 3.3 Inclusion and exclusion criteria

For inclusion the published article on HHT-associated brain AVMs must:

1) Report of at least one case of surgically treated brain AVM related to HHT
2) Article published in English

Articles reporting only nonsurgical treatment of HHT-associated brain AVMs and non-English articles were excluded.

### 3.4 Statistical analysis

Fisher's exact test was used to determine whether there was a statistically significant difference in the rate of permanent neurological morbidity between the groups of patients operated on for HHT-associated brain AVM and for sporadic brain AVM.

Sensitivity analyses were performed to assess the robustness of the pooled estimate. First, a leave-one-out analysis was conducted by sequentially excluding each study and recalculating the pooled permanent morbidity rate and the Fisher's exact test comparison against each reference cohort. This approach identifies studies with disproportionate influence on the overall result. Second, the analysis was restricted to studies rated as high quality (≥8/10) on the JBI Critical Appraisal Checklist to evaluate whether study quality affected the pooled estimate. The 95% exact binomial confidence interval for the pooled permanent morbidity rate was calculated.

## 4 Results

The list of articles included in this review and the extracted data are presented in Table 1. Eighteen articles fulfilled the inclusion criteria [Izhar et al., 2026; Frantzias et al., 2024; Beslow et al., 2024; Kilian et al., 2023; Azma et al., 2022; Woodall et al., 2019; Meybodi et al., 2018; Gamboa et al., 2018; Rattani et al., 2017; Yang et al., 2016; Woodall et al., 2014; Du et al., 2007; Willemse et al., 2000; Haitjema et al., 1995; Kikuchi et al., 1994; Aesch et al., 1991; Lesser et al., 1986; Waller et al., 1976]. Twelve of them reported both number of operated patients as well as number of operated brain AVMs [Izhar et al., 2026; Frantzias et al., 2024; Beslow et al., 2024; Woodall et al., 2019; Meybodi et al., 2018; Gamboa et al., 2018; Du et al., 2007; Haitjema et al., 1995; Kikuchi et al., 1994; Aesch et al., 1991; Lesser et al., 1986; Waller et al., 1976], five papers reported only the number of operated patients [Kilian et al., 2023; Azma et al., 2022; Rattani et al., 2017; Yang et al., 2016; Willemse et al., 2000], one paper reported only the number of operated AVMs [Woodall et al., 2014].

**Table 1:** List of articles reporting surgically treated HHT-associated brain AVMs with corresponding data.

Authors, year	Nr of included patients/Nr of included bAVMs	Nr of operated patients/Nr of operated bAVMs	Nr of patients operated for ruptured bAVMs/for unruptured bAVMs	Surgery as a part of combined treatment* (Nr of patients)	Nr of patients suffering transient surgical complications	Nr of patients suffering permanent surgical complications
Izhar et al. (2026)	26/39	22/25	0/22	0	NA	7
Frantzias et al. (2024)	20/28	20/23	3/17	2	4	1
Beslow et al. (2024)	40/54	7/7	3/4	1	NA	NA
Kilian et al. (2023)	357/NA	109/NA	32/77	32	NA	NA
Azma et al. (2022)	20/23	8/NA	2/6	2	NA	1
Woodall et al. (2019)	14/27	12/13	2/10	2	1	0
Meybodi et al. (2018)	41/53	19/20	1/18	7	4	0
Gamboa et al. (2018)	39/78	22/26	4/18	4	NA	NA
Rattani et al. (2017)	2/NA	2/NA	0/2	0	0	0
Yang et al. (2016)	12/23	2/NA	0/2	0	0	0
Woodall et al. (2014)	NA/13	NA/6	NA	1	NA	NA
Du et al. (2007)	1/4	1/1	1/0	0	1	0
Willemse et al. (2000)	22/28	3/NA	0/3	0	NA	NA
Haitjema et al., 1995	10/NA	1/1	0/1	0	NA	0
Kikuchi et al. (1994)	4/6	1/1	1/0	0	0	0
Aesch et al. (1991)	1/2	1/2	1/1	0	0	0
Lesser et al. (1986)	2/2	2/2	1/1	0	0	0
Waller et al. (1976)	3/3	1/1	0/1	0	NA	NA

HHT - Hereditary hemorrhagic telangiectasia; bAVMs - brain arteriovenous malformations; Nr – number; NA – not available; *Combined treatment = combination of bAVM resection with radiosurgery and/or embolization.

### 4.1 Study quality assessment

JBI Critical Appraisal scores ranged from 5/10 to 10/10 (Supplementary Table 1). Six studies were rated as high quality (≥8/10): Yang et al. (10/10), Frantzias et al. (10/10), Meybodi et al. (8/10), Woodall et al., (2019) (8/10), Azma et al. (8/10), and Izhar et al. (8/10). Six studies were rated as low quality (≤6/10): Lesser et al. (6/10), Aesch et al. (6/10), Kikuchi et al. (6/10), Du et al. (6/10), Rattani et al. (6/10), and Haitjema et al. (5/10). The most common methodological limitations were unclear consecutive inclusion of participants and unclear completeness of inclusion (which are inherent to retrospective case series). No study scored “No” on any item; all weaknesses were rated as “Unclear” rather than definitively absent.

### 4.2 Postoperative deficits after resections of HHT-associated AVMs

Data on the presence/absence of neurological outcome were reported in 12 out of 18 papers.

Izhar et al. conducted a retrospective analysis of 26 HHT patients, 22 of whom underwent surgery for 25 unruptured brain AVMs. The overall long-term neurological status was improved in two patients (9%), unchanged in 13 patients (59%) and worsened in seven patients (32%). Of note, 91% of surgical patients (20/22) achieved good functional outcome (mRS 0–1) at last follow-up; only two patients had mRS scores of 2 and 3, indicating that most instances of neurological worsening were mild (i.e., mRS 0 to mRS 1). Four patients with 14 AVMs underwent SRS, permanent neurological worsening was observed in one patient after SRS (25%), three patients remained unchanged [Izhar et al., 2026].

Frantzias et al. retrospectively reviewed 20 HHT patients in whom 23 brain AVMs were resected, most of them were Spetzler-Martin grade I and II. Postoperative complications were detected in five patients – three patients had visual field deficit - two of them improved in long-term follow up. One patient had transient motor deficit and another had transient speech and motor deficit [Frantzias et al., 2024].

In the series reported by Azma et al. eight patients were operated, one patient developed cerebellar infarction after surgery (neurological sequelae of the infarction were not specified) [Azma et al., 2022].

In the series reported by Woodall et al., there were no permanent neurological deficits as a consequence of surgical treatment of brain AVMs in 12 patients with HHT. However, one patient developed upper limb monoparesis after surgery for a small AVM in the primary sensory cortex, which resolved completely at the first follow-up visit [Woodall et al., 2019].

Meybodi et al. retrospectively reviewed for changes in functional status 19 patients (in whom 20 AVMs were resected) from the database of the Brain Vascular Malformation Consortium HHT Project. The patients were recruited at eight different neurosurgical centers. Three patients suffered from early postoperative neurological complications that resolved within one month after surgery - transient sensorimotor deficits in one frontal and one parietal AVM and transient dysmetria in a cerebellar AVM. One patient with a left frontal AVM had an associated aneurysm that ruptured intraoperatively and caused hemiparesis and dysphasia; these neurological deficits rapidly improved over the following months, and the patient was completely symptom-free at the first long-term follow-up visit [Meybodi et al., 2018].

Du et al. presented a case of 26-day-old male with a family history of HHT1 who suffered a left frontoparietal intraparenchymal hemorrhage; cerebral angiography revealed multiple AVMs. A left parietal AVM was resected five months after the stroke. Immediate postoperative right hemiparesis completely resolved after four months [Du et al., 2007].

In the remaining six articles that reported data on surgical complications, the absence of postoperative neurological sequelae was reported [Rattani et al., 2017; Yang et al., 2016; Haitjema et al., 1995; Kikuchi et al., 1994; Aesch et al., 1991; Lesser et al., 1986]. Postoperative mortality following HHT-associated AVM surgery was not reported in any article.

### 4.3 Comparison of permanent postoperative neurological sequelae after surgeries for HHT-associated AVMs versus surgeries for sporadic AVMs

The overall incidence of new permanent neurological morbidity among 91 operated HHT patients (included in the 12 articles which reported functional outcomes after resections of brain AVMs) was 9.9% (nine patients).

For the purpose of comparing the incidence of permanent new neurological morbidity after resection of cerebral AVMs associated with HHT with the incidence of permanent new neurological morbidity after resection of unselected sporadic cerebral AVMs, three reference cohorts of unselected sporadic cerebral AVMs were selected: 1) a large-scale systematic review and meta-analysis of 142 cohorts totaling 13,698 patients with a subgroup of 2549 patients treated with microsurgery authored by van Beijnum et al.- postoperative complications leading to new permanent neurological deficit or death occurred in a median of 7.4% of the patients after microsurgery [van Beijnum et al., 2011]; 2) a systematic review of 12 studies within the subgroup of 711 surgically treated patients (with or without prior embolization) authored by Baptista et al. - the incidence of postoperative neurological deficit was 11.67% (n = 83) [Baptista et al., 2025]; 3) a consecutive single-surgeon series of 288 microsurgical cases authored by Schramm et al. - permanent neurological changes were observed in 12.2% of cases (n = 35) [Schramm et al., 2017].

We did not find a statistically significant difference between the incidence of new permanent postoperative neurological morbidity in the set of 91 patients with HHT-associated AVMs which we compiled from the published literature, and selected large cohorts of sporadic unselected brain AVMs reported by van Beijnum et al. [2011] (P = 0.4136), Baptista et al. [2025] (P = 0.7281), and Schramm et al. [2017] (P = 0.7076).

### 4.4 Spetzler-Martin grade distribution

Spetzler-Martin grade was identifiable in 73 of the operated HHT-associated AVMs: 46 (63.0%) were SM grade I, 22 (30.1%) were SM grade II, three (4.1%) were SM grade III, and two (2.7%) were SM grade IV; no SM grade V AVMs were identified. Overall, 68 of 73 AVMs (93.2%) were low-grade (SM I–II). This distribution was significantly different from that reported in large unselected sporadic AVM surgical series: Schramm et al. [2017] reported 58.0% low-grade AVMs (167/288; P < 0.0001, Fisher's exact test), and Ren et al. [2017] reported 53.7% low-grade AVMs (239/445; P < 0.0001). The predominance of low-grade lesions in the HHT cohort is consistent with published meta-analytic data showing that 86.9% of HHT-associated brain AVMs are SM grade ≤ II [Brinjikji et al., 2017].

### 4.5 Sensitivity analyses

The pooled permanent neurological morbidity rate of 9.9% (9/91; 95% CI: 4.6–18.0%) was substantially influenced by a single study. Leave-one-out analysis revealed that exclusion of the study by Izhar et al. [2026] reduced the pooled rate from 9.9% (9/91) to 2.9% (2/69), while exclusion of any other individual study changed the pooled rate by ≤ 2.6 percentage points (range: 9.6%–12.5%). The series presented by Izhar et al. [2026] contributed seven of nine total permanent deficit events (78%) despite comprising only 24% of the pooled cohort. When the study by Izhar et al. [2026] was excluded, the HHT cohort rate (2.9%) was significantly lower than the neurological morbidity rates reported by Baptista et al. [2025] (11.67%; P = 0.018) and Schramm et al. [2017] (12.2%; P = 0.023), but not significantly different from van Beijnum et al. [2011] (7.4%; P = 0.174).

When the analysis was restricted to the six studies rated as high quality on the JBI Critical Appraisal Checklist (83 patients, nine events), the pooled permanent morbidity rate was 10.8%, which was similar to the full-cohort estimate of 9.9%, suggesting that the overall result was robust to study quality exclusions. Notably, all nine permanent deficit events occurred in high-quality studies; the six low-quality studies (eight patients total) reported no permanent deficits.

## 5 Discussion

Given the complexity of HHT treatment, it is of utmost importance that neurosurgeons as well as patients have access to all available relevant data on the risks of individual treatment modalities. During the preparation of the treatment plan for the presented patient, we could not identify any article that would systematically evaluate the long-term outcomes of patients operated on for HHT-associated brain AVMs. Our systematic review of literature fills the aforementioned gap at least partially.

The long-term outcomes of resections of HHT-associated AVMs seem not to differ from resections of sporadic cases. When we compared the incidence of new permanent postoperative deficits in the group of sporadic AVMs [Baptista et al., 2025; Schramm et al., 2017; van Beijnum et al., 2011] and in the group of HHT-associated brain AVMs we collected from the literature [Izhar et al., 2026; Frantzias et al., 2024; Azma et al., 2022; Woodall et al., 2019; Meybodi et al., 2018; Rattani et al., 2017; Yang et al., 2016; Du et al., 2007; Haitjema et al., 1995; Kikuchi et al., 1994; Aesch et al., 1991; Lesser et al., 1986], no statistical difference was found. However, the number of published patients operated on for HHT-associated AVMs is surprisingly low, and cases with reported permanent neurological outcome are even more sparse. A formal meta-analysis was not feasible because of the small number of studies reporting the outcome of interest and the very low event count (nine events across 12 case series), with several studies contributing zero events. Consequently, study-level effect sizes could not be estimated reliably, a forest plot was not constructed, and heterogeneity (I^2^, Q-statistic) and publication bias could not be formally assessed. It must be emphasized that the absence of a statistically significant difference between the two groups should not be interpreted as evidence of equivalence - with nine events among 91 patients in the HHT cohort, the study is substantially underpowered to detect clinically meaningful differences. The 95% exact binomial confidence interval for the pooled permanent morbidity rate of 9.9% is wide (4.6–18.0%), reflecting considerable imprecision. Published data indicate that the vast majority of meta-analyses involving rare events are underpowered [Jia et al., 2021]. Hence, the present findings should be considered descriptive and hypothesis-generating rather than confirmatory.

The sensitivity analysis demonstrated that the pooled permanent neurological morbidity estimate is fragile and heavily dependent on a single study (Izhar et al.), which contributed 78% of all permanent deficit events while comprising only 24% of the pooled cohort. This finding underscores the need for additional prospective data to establish a more accurate estimate. Notably, when the study by Izhar et al. was excluded, the HHT cohort's morbidity rate (2.9%) was significantly lower than that of unselected sporadic AVMs (Baptista et al.: P = 0.018; Schramm et al.: P = 0.023), which is consistent with the predominantly low-grade composition of HHT-associated AVMs (93.2% SM I–II). Whether the higher morbidity rate reported by Izhar et al. [2026] reflects true differences in surgical complexity, patient selection, institutional factors, or chance variation in a small sample cannot be determined from the available data. The analysis restricted to high-quality studies (JBI ≥8/10; 83 patients, nine events) yielded a pooled rate of 10.8%, similar to the full-cohort estimate, suggesting robustness to study quality exclusions.

It is necessary to emphasize that although HHT status was not identified as an independent risk factor for surgical morbidity or specific postoperative complications in the literature, and although we have found no differences between the compared groups it does not mean that a difference does not exist. HHT is caused by germinal mutations in genes encoding components of the TGF-β/BMP signaling pathway (*ENG, ACVRL1/ALK1, SMAD4*), resulting in impaired function of endothelial cells. This leads to increased endothelial proliferation and defective arteriovenous differentiation, with a propensity for vessel wall fragility and abnormal hemodynamics [Drapé et al., 2022; Derdeyn et al., 2017; Kalailingam et al., 2025]. Sporadic brain AVMs are commonly associated with somatic mutations in the RAS/MAPK pathway, leading to focal vascular dysplasia without the systemic endothelial dysfunction seen in HHT [Drapé et al., 2022; Derdeyn et al., 2017; Kalailingam et al., 2025]. Whether these genetic and pathophysiological differences between both AVM types translate into different perioperative risks and permanent functional outcomes is currently unknown. However, it is a known fact that HHT-associated brain AVMs differ from sporadic brain AVMs in terms of multiplicity, size, and Spetzler-Martin grade, which may influence surgical decision-making and risk assessment [Yang et al., 2016; Willemse et al., 2000; Woodall et al., 2014; Derdeyn et al., 2017]. Interestingly, some studies have reported spontaneous regression of AVMs in patients with HHT [Cloft, 2002; Leung et al., 2006] and a lower risk of rupture [Yang et al., 2016] - findings that may support a more conservative treatment approach. In contrast, others advocate more aggressive approach, arguing that the rupture risk is indeed similar in HHT-associated and sporadic AVMs [Gamboa et al., 2018; Woodall et al., 2019].

The different SM grade distribution between HHT-associated and sporadic AVMs has important implications for interpreting the morbidity comparison - the unselected sporadic comparator cohorts include significantly higher proportion of high-grade lesions (SM III–V) in which poor outcome is significantly more frequent [Derdeyn et al., 2017]. This compositional difference means that the comparison of the HHT cohort against unselected sporadic series is inherently conservative: the sporadic cohorts' morbidity rates are inflated by the inclusion of higher-risk lesions. Despite more favorable grade distribution in the HHT cohort, its pooled permanent morbidity rate (9.9%) falls within the range reported for unselected sporadic AVMs (7.4–12.2%), which could suggest that HHT-associated AVMs may carry higher surgical risk than their grade distribution alone would predict; however, this observation is heavily influenced by a single study (Izhar et al., 2026), and the small sample size precludes definitive conclusions. Grade-specific surgical outcomes could not be extracted from the majority of included studies, precluding a grade-stratified pooled analysis; future studies should report SM grade-specific outcomes to enable more precise comparisons.

Importantly, given the unpredictable behavior of AVMs in HHT patients (spontaneous regression versus new AVM development) serial imaging should be considered. While the use of MRI is recommended as a primary screening tool for cerebral AVMs in HHT patients by current HHT treatment guidelines [Faughnan et al., 2020], the optimal methods and frequency for surveillance of already known untreated HHT-associated brain AVMs are not well established in the medical literature. The situation is similar in sporadic AVMs – there are few data for the utility of imaging for long-term surveillance for untreated brain AVMs [Derdeyn et al., 2017]. While DSA has the highest degree of both spatial and temporal resolution of all diagnostic imaging modalities, due to the ionizing radiation risks there are efforts to advance MR techniques, particularly in children [Derdeyn et al., 2017]. On the other hand, DSA can demonstrate detailed angioarchitectural features of both sporadic and HHT-associated AVMs that connote a higher risk of future rupture that are often not discernible on MR imaging, including feeding artery aneurysms, nidal aneurysms, and venous outflow stenosis [Vella et al., 2020].

Stereotactic radiosurgery (SRS) represents an important alternative treatment modality for HHT-associated brain AVMs. Three recent studies provide outcome data. González et al. reported on eight patients with 14 AVMs treated with SRS, achieving obliteration in 11 AVMs (79%) at a median follow-up of 112.75 months. In three AVMs (followed-up 15.2, 48.3 and 430 months) obliteration was not observed, time to obliteration in the 11 obliterated AVMs was 35.83 months. One post-SRS hemorrhage was observed, all patients maintaining favorable functional status (mRS 0–1) at last follow-up [González et al., 2024]. In a larger multicenter series, Akiyama et al. included 31 patients with 63 AVMs, reporting cumulative post-SRS obliteration rates of 25.3% at three years, 61.6% at five years, and 81.2% at seven years (four AVMs underwent secondary SRS for a lack of obliteration after 45 months); neurological status remained unchanged or improved in 90.3% of patients, while 9.7% experienced worsening [Akiyama et al., 2026]. Izhar et al. reported SRS outcomes in four patients with 14 AVMs, with 80% achieving complete obliteration at a median follow-up of 5.9 years; permanent neurological worsening occurred in one patient (25%) [Izhar et al., 2026]. Notably, the median time to obliteration after SRS in HHT-associated AVMs appears longer than in sporadic AVMs, possibly reflecting differences in angioarchitecture [Akiyama et al., 2026].

This prolonged latency period — during which hemorrhage risk persists — represents a key trade-off compared to microsurgical resection, which offers immediate AVM elimination. The situation is further complicated by the fact that while successful resection of one HHT-associated AVM eliminates its hemorrhage risk, patients remain at risk for new lesion development, hence SRS must be considered as a viable treatment option. Treatment decisions should therefore be individualized, weighing the immediate but higher acute risk of surgery against the delayed but potentially lower acute morbidity of SRS, while also considering patient preferences, AVM multiplicity, and the possibility of de novo AVM formation in HHT patients.

Several limitations must be acknowledged. First, the pooled cohort of 91 patients represents all published cases of surgically treated HHT-associated brain AVMs with reported permanent neurological outcomes available in the world literature; the small sample size and low number of events (nine permanent deficits) result in wide confidence intervals and limited statistical power to detect clinically meaningful differences. Second, all included studies are retrospective case series with inherent selection and reporting biases; six of twelve studies scored ≤ 6/10 on the JBI Critical Appraisal Checklist, and the review protocol was not prospectively registered. Third, the pooled estimate is fragile and heavily influenced by a single study (Izhar et al. [2026]Izhar et al. [2026]), which contributed 78% of all permanent deficit events. In addition, while reporting seven patients who permanently worsened postoperatively, they also stated that only two of these patients had mRS > 1 (mRS = 2 and mRS = 3), suggesting that most instances of worsening were mild. Fourth, the comparison with sporadic AVM cohorts relies on external benchmark rates rather than a matched control group, and the comparator cohorts include significantly more higher-grade lesions that inflate their morbidity rates relative to the predominantly low-grade HHT cohort. Fifth, SM grade-specific surgical outcomes could not be extracted from the majority of included studies, precluding grade-stratified analysis. Sixth, heterogeneity in outcome definitions, follow-up duration, and reporting completeness across studies limits the precision of the pooled estimate. A formal meta-analysis with forest plot, heterogeneity assessment (I^2^, Q-statistic), and publication bias evaluation was not feasible due to the small number of events. Despite these limitations, this systematic review represents the most comprehensive synthesis of available surgical outcome data for HHT-associated brain AVMs and provides a foundation for future prospective studies.

The illustrative case presented in this study exemplifies the clinical context in which the data from this systematic review are most needed. The patient declined stereotactic radiosurgery due to its delayed effect and his personal experience with the death of his father from HHT-associated AVM hemorrhage. The patient's outcome (transient aphasia fully resolved at one month, complete AVM resection confirmed on postoperative imaging) is consistent with the relatively favorable surgical outcomes observed in this review. While individual cases cannot be generalized, this scenario illustrates that microsurgical resection can be a reasonable option in carefully selected HHT patients who require or prefer immediate definitive treatment.

## 6 Conclusion

The number of published cases of resected HHT-associated brain AVMs remains limited, precluding firm conclusions. The available data did not demonstrate a statistically significant difference in permanent neurological morbidity between HHT-associated and sporadic AVM resections; however, the small sample size and the disproportionate influence of a single study on the pooled estimate preclude definitive conclusions, and these findings should be considered hypothesis-generating. Treatment decisions should be individualized, considering Spetzler-Martin grade, patient age, lesion multiplicity, hemorrhage risk stratification, patient preferences, and the availability of alternative treatments including stereotactic radiosurgery. Prospective multicenter registries with standardized outcome reporting are needed to enable grade-stratified outcome comparisons and to better define optimal management strategies for HHT-associated brain AVMs.

## Informed consent

Patient's written consent for publication has been obtained.

## Author contributions

Ján Buvala – concept, data collection, analysis, drafting manuscript, reviewing final manuscript.

Christopher S. Ogilvy – concept, reviewing final manuscript.

Juraj Šteňo – concept, reviewing final manuscript.

Juraj Klepoch jr. – data collection, reviewing final manuscript.

Vladimír Javorka – concept, reviewing final manuscript.

Michaela Jezberová – concept, reviewing final manuscript.

Peter Jankovič – concept, reviewing final manuscript.

Parvez M. Shaikh – concept, analysis, reviewing final manuscript.

Andrej Šteňo – concept, data collection, analysis, drafting manuscript, reviewing final manuscript.

## Ethical approval

Obtaining ethical approval is not necessary for conducting a systematic review; all applicable international, national, and institutional guidelines for the care and use of the patient's anonymized clinical data were followed.

## Data availability

All data analyzed in this review are available in the cited published articles and in Supplementary Table 1.

## Declaration of generative AI and AI-assisted technologies in the manuscript preparation process

During the preparation of this work, the authors used OpenEvidence (OpenEvidence Inc.) in order to critically review the logical flow of arguments, identify potential structural weaknesses, and refine the phrasing of standard methodological frameworks. After using this tool, the authors reviewed and edited the content as needed and take full responsibility for the content of the published article.

## Funding

This work was supported by a grant from the Slovak Agency for Research and Development (APVV; grant no. 23-0577).

## Declaration of competing interest

Andrej Šteňo is a consultant for Brainlab SE, Munich, Germany, the manufacturer of the 3D ultrasound system used for intraoperative imaging presented in this article; Brainlab SE did not provide any financial support in connection with this publication. The other authors declared no potential conflicts of interest with respect to the research, authorship, and/or publication of this article.
