# Supplementary material for: Permanent neurological morbidity after microsurgical resection of hereditary hemorrhagic telangiectasia-associated brain arteriovenous malformations: a systematic review and illustrative case
Source: Brain Spine. 2026 Sep 22;6:107455. doi: 10.1016/j.bas.2026.107455 (PMC13634297; doi:10.1016/j.bas.2026.107455)
Supplement: Supplementary file 1 [file mmc1.docx]

**Supplementary Table 1** - Methodological quality assessment of the included studies using the Joanna Briggs Institute (JBI) Critical Appraisal Checklist for Case Series [Aromataris et al., 2024]

| **Authors, Year** | **Q1** | **Q2** | **Q3** | **Q4** | **Q5** | **Q6** | **Q7** | **Q8** | **Q9** | **Q10** | **Total score (Yes/10)** |
| --- | --- | --- | --- | --- | --- | --- | --- | --- | --- | --- | --- |
| **Lesser et al., 1986** | N/A | Yes | Yes | N/A | N/A | Yes | Yes | Yes | Yes | N/A | 6/10 |
| **Aesch et al., 1991** | N/A | Yes | Yes | N/A | N/A | Yes | Yes | Yes | Yes | N/A | 6/10 |
| **Kikuchi et al., 1994** | Unclear | Yes | Yes | Unclear | Unclear | Yes | Yes | Yes | Yes | N/A | 6/10 |
| **Haitjema et al., 1995** | Unclear | Yes | Yes | Unclear | Unclear | Unclear | Unclear | Yes | Yes | Yes | 5/10 |
| **Du et al., 2007** | N/A | Yes | Yes | N/A | N/A | Yes | Yes | Yes | Yes | N/A | 6/10 |
| **Yang et al., 2016** | Yes | Yes | Yes | Yes | Yes | Yes | Yes | Yes | Yes | Yes | 10/10 |
| **Rattani et al., 2017** | N/A | Yes | Yes | N/A | N/A | Yes | Yes | Yes | Yes | N/A | 6/10 |
| **Meybodi et al., 2018** | Yes | Yes | Yes | Unclear | Unclear | Yes | Yes | Yes | Yes | Yes | 8/10 |
| **Woodall et al., 2019** | Yes | Yes | Yes | Unclear | Unclear | Yes | Yes | Yes | Yes | Yes | 8/10 |
| **Azma et al., 2022** | Yes | Yes | Yes | Yes | Unclear | Yes | Yes | Unclear | Yes | Yes | 8/10 |
| **Frantzias et al., 2024** | Yes | Yes | Yes | Yes | Yes | Yes | Yes | Yes | Yes | Yes | 10/10 |
| **Izhar et al., 2026** | Yes | Yes | Yes | Unclear | Unclear | Yes | Yes | Yes | Yes | Yes | 8/10 |

Q1-10 – Question 1-10, N/A - Not Applicable

The methodological quality of included studies was assessed independently by two authors (J.B. and A.Š.) using the 10-item Joanna Briggs Institute Critical Appraisal Checklist for Case Series [Aromataris et al., 2024]. Studies were classified as high (≥8/10), moderate (7/10), or low quality (≤6/10), with disagreements resolved by consensus.
